# Upcycling Orange-Based Waste into Functional CNCs for Greener *L*-Lactide Ring-Opening Polymerization

**DOI:** 10.3390/polym17192605

**Published:** 2025-09-26

**Authors:** Adrián Leonés, Cayetano Sánchez-Solís, Asier Medel, Maria P. García-Aparicio, Marta E. G. Mosquera, Valentina Sessini

**Affiliations:** 1 Departamento de Química Orgánica y Química Inorgánica, Facultad de Ciencias, Universidad de Alcalá, Ctra. Madrid-Barcelona Km. 33.6, Madrid, 28805 Alcalá de Henares, Spain; cayetano.sanchez@edu.uah.es (C.S.-S.); asier.medel@uah.es (A.M.); 2 Advanced Biofuels and Bioproducts Unit, Renewable Energy Division, CIEMAT, 28040 Madrid, Spain; m.delprado@ciemat.es

**Keywords:** sulfuric acid, phosphoric acid, hydrochloric acid, acid hydrolysis, citrus peels, polylactide

## Abstract

This study demonstrates the valorization of orange peel waste as a sustainable feedstock for the production of cellulose nanocrystals (CNCs). Compositional analysis revealed a cellulose content up to 10.0% in the raw material. After performing the alkaline/peroxide treatment, CNCs were isolated via acid hydrolysis. Different inorganic acids were compared, namely sulfuric, phosphoric, and hydrochloric acids at low molar concentrations. The resulting CNCs showed distinct morphological and physicochemical properties, with sulfuric acid treatment yielding the highest crystallinity index (TCI) of 0.86 under conditions of 3.0 mol/L, 80 °C, and 225 min. Additionally, the presence of sulfate or phosphate groups significantly influenced the thermal degradation behavior and the inorganic residue content in the obtained CNCs. Finally, the CNCs were successfully tested as co-initiator for lactide ring-opening polymerization. The results show that the molecular weights of the resulting polylactide varied depending on the CNC dispersion. This work supports the use of orange peel waste as a bio-source for CNC production and their potential application as a co-initiator in the synthesis of polyesters.

## 1. Introduction

The growing demand for sustainable materials is driving the development of alternatives to traditional plastics, particularly to reduce the environmental pollution associated with their production and disposal [1]. In this context, the revalorization of agro-industrial organic waste as a source of biobased materials has gained increasing attention. The juice industry alone generates an estimated 25 million tons of residues annually [1], with orange (*Citrus sinensis*) being the most processed fruit, generating around 16 million tons of waste every year [2]. These organic wastes represent a promising renewable feedstock for biopolymer production, contributing to the development of sustainable materials in the context of a greener circular economy [3].

Among the different biopolymers, cellulose is the most abundant natural polymer, and it is characterized by its low carbon footprint [4]. In particular, cellulose nanocrystals (CNCs) isolated from natural sources are usually used as filler for polymer nanocomposites due to their low toxicity [5], high-aspect-ratio [6], and ability to enhance the mechanical properties of polymeric matrices [7]. Cellulose constitutes the main component of orange waste, but its extraction requires multiple purification steps. Usually, an alkali pulping treatment is applied to remove non-cellulosic components, followed by acid hydrolysis treatment to isolate CNCs from amorphous fractions of the kraft cellulose. The acid hydrolysis of cellulosic materials involves the cleavage of *β*-1,4 glycosidic bonds in the cellulose structure [8]. This hydrolysis primarily affects the amorphous regions surrounding the crystalline ones because they are less densely packed. This reaction can be carried out with different inorganic acids and usually requires high molar concentration, allowing for the isolation of CNCs of different morphologies and functionalities, such as whisker shapes [9] or micro/nanofibrils [10]. Moreover, the use of different inorganic acids introduces different charged groups to the surface of CNCs, affecting the CNC–CNC interactions and, consequently, their properties [8].

Several studies have demonstrated the isolation of CNCs from orange-based wastes. For example, Mantovan et al. obtained cellulose-based materials from orange bagasse using sulfuric acid (60% *w*/*w*) combined with autoclaving and ultrasonication steps, and they reported a cellulose recovery of 65.3–100% [11]. Moreover, Palaniappan et al. used hydrochloric acid (50%, 1:3 *v*/*v*) to isolate microcrystalline cellulose from sweet orange peel fruit waste, obtaining cellulose nanocrystals with a size of 9.63 nm and a crystallinity index of 72.54% [12]. However, comparative studies using different inorganic acids at varying molarity and reaction conditions remain limited to other cellulose sources, such as cotton [13]. Therefore, further investigation is needed on orange-based waste materials.

CNCs exhibit abundant hydroxyl groups, which enable their use as substrates in surface-initiated ring-opening polymerization (SI-ROP) reactions. Their nucleophilic nature allows them to act as co-initiators in polymerization processes such as the synthesis of poly-*L*-lactide (PLLA), a biobased thermoplastic which is usually obtained by the ROP of lactide, a cyclic diester derived from lactic acid [14]. This polymerization usually requires the use of efficient initiators and metal-based catalysts to achieve high conversion and desired tacticity [15]. Although tin-based catalysts like stannous octanoate, [Sn(Oct)_2_], are industrially prevalent, concerns over their toxicity have prompted the search for alternative main group metal catalysts [15], such as potassium [16,17] or magnesium [18,19]. In our group, we have previously reported a diphenoxyimine five-coordinated aluminum complex, [AlMeL_2_], (L = N-(2,6-diisopropylphenyl)-phenoxyimine), which is an active ROP catalytic for different monomers, such as rac-*β*-butyrolactone (BBL) [20], *L*-lactide, and ε-caprolactone [21]. In particular, this [AlMeL_2_] complex allowed for the obtention of polymers with narrow dispersity and controlled molecular weight when used in the presence of benzyl alcohol (BnOH) as a co-initiator [21]. Given the reactivity of CNC hydroxyl groups, this aluminum-based system is a strong candidate for performing surface-initiated ring-opening polymerization from CNC surfaces. Organocatalysts such as 1,5,7-triazabicyclo [4.4.0] dec-5-ene (TBD), and *N,N*-dimethyl aminopyridine (DMAP), have also been used to study the SI-ROP of ε-caprolactone [22] and lactide [23] from cellulose; however, there are few examples of metal catalysts used for this purpose.

In this study, we investigate the revalorization of orange peel waste for the production of CNCs and their subsequent application for the synthesis of PLLA via aluminum-catalyzed ROP of *L*-lactide. Orange waste was firstly subjected to an alkaline/peroxide treatment, followed by acid hydrolysis using diluted H_3_PO_4_, HCl, or H_2_SO_4_ under mild conditions. The resulting cellulosic materials were characterized in terms of morphology and thermal and chemical properties. Finally, the CNCs were tested as co-initiators in the SI-ROP of *L*-lactide using the [AlMeL_2_] catalysts. The dispersion of CNCs into the polymerized PLLA was also investigated, along with the thermal properties of the resulting nanocomposite. This work aims to contribute to the sustainable synthesis of PLLA while promoting the revalorization of orange waste derived from the industrial activity.

## 2. Materials and Methods

### 2.1. Materials

Orange peel waste from citric beverage production was kindly provided by Refrescos Iberia S.A.U (46780 Oliva, Valencia, Spain).

All the reagents were used as received without further purification. Sulfuric acid (H_2_SO_4_, 98%, 98.07 g/mol) was purchased from Labkem (Barcelona, Spain), chlorohydric acid (HCl, 37%, 36.46 g/mol) from PanReac AppliChem (Barcelona, Spain), and phosphoric acid (H_3_PO_4_, 85%, 97.99 g/mol) from Glenthan Life Sciences (Corsham, UK). *L*-lactide (>98%) was purchased from TCI Chemicals (Tokyo, Japan) and purified by recrystallization in toluene, followed by sublimation at 100 °C. The purified monomer was stored in a glovebox. AlMe_3_ (2.0 M in toluene), 2-hydroxybenzaldehyde and 2,6-diisopropylaniline were purchased from Sigma-Aldrich (St. Louis, MO, USA). All the reagents employed in the synthesis of the preligand, and the aluminum-based compound was commercially obtained and used without further purification.

### 2.2. Compositional Analysis of Biomass

Orange peel waste was manually homogenized, and its moisture content was determined by measuring the weight loss after oven-drying at 105 °C until a constant weight was achieved. For chemical characterization, a representative sample of orange peel waste was dried at 40 °C in a convection oven for 3 days. The dried biomass was subsequently ground and sieved to a particle size of 1 mm using a centrifugal mill (Retsch ZM200, Retsch GmbH, Haan, Germany). Compositional analysis was carried out following standardized laboratory protocols developed by the National Renewable Energy Laboratory (NREL, Golden, CO, USA), including Technical Report NREL/TP-510-42620, 42618, 42619, 42620, and 42622 [24], to determine the extractives, structural components, and inorganic material of the biomass. The determination of the main structural components of the biomass (such as glucan, cellulose, hemicellulose, and acid insoluble residue) was conducted in a two-step acid hydrolysis on extracted-free material and subsequent quantification of the monomeric sugars using high-performance liquid chromatography (HPLC). The concentrations of sugar (sucrose, glucose, xylose, fructose, galactose, arabinose, and mannose) on the water extractives fraction and the acid-hydrolyzed samples were determined using a Waters Alliance 2695 system (Waters, Milford, MA, USA) equipped with a 2414 refractive index detector and a CarboSep CHO-782 column (Transgenomic, Omaha, NE, USA). The column operated at 70 °C, with ultrapure water as the mobile phase at a flow rate of 0.5 mL/min. All analyses were conducted in triplicate to ensure reproducibility.

Based on these data, the contents of cellulose, hemicellulose, and total lignin were estimated and expressed as a percentage of the dry weight of the extractive-free sample.

### 2.3. Alkaline/Peroxide Treatment of Orange Peel Waste

Before the acid hydrolysis, the orange peel waste was subjected to an initial alkaline process with NaOH solution (2 wt%). After 1 h at 55 °C under continuous stirring, the solid obtained was filtered using a metallic filter and treated with H_2_O_2_ to further remove non-cellulosic material and bleach the residue.

### 2.4. Isolation of Cellulosic Materials by Acid Hydrolysis

**Scheme 1:**
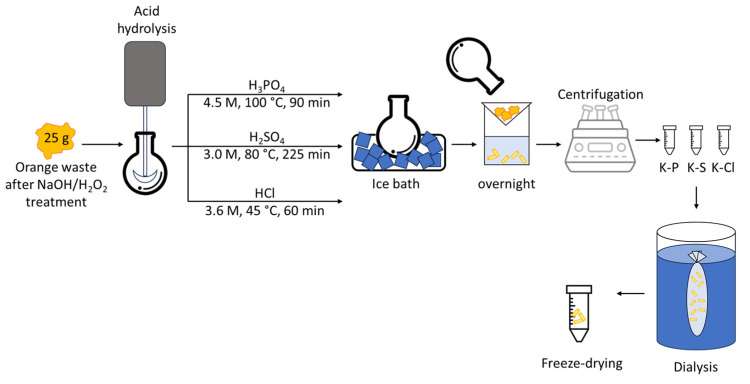
Schematic representation of the different acid hydrolysis treatments.

After the alkaline/peroxide treatment, CNCs were isolated using dilute acid hydrolysis under three different conditions. In each case, 25 g of alkaline/peroxide-treated orange waste (K-cellulose) was weighed in a round-bottom flask and different acid solutions were immediately added. The reactions were mechanically stirred at different temperatures and durations, as summarized in Table 1. Subsequently, the mixture was cooled in an ice bath, and the residues were filtered using a metallic filter. Suspensions were allowed to settle overnight before being centrifugated to recover the cellulosic material (Beckman Avanti J-25, Brea, CA, USA) and neutralize it. A total of three centrifugations (15,000–24,500 rpm, 20 °C) were carried out for each sample using deionized water, achieving final pH levels of 3.7, 4.7 and 0.7 for K-P, K-S, and K-Cl, respectively (see Scheme 1).

Dialysis purification (Spectra/Por 6 membrane, 1 kDa) was performed to neutralize the material dispersion. Aqueous suspensions of each sample were dialyzed against deionized water, with the water replaced daily until reaching neutral pH. In particular, K-P and K-S showed final pH values of 7.5 and 6.9, respectively, after 6 days of dialysis, and K-Cl showed a pH of 6.3 after 10 days.

Finally, the neutralized aqueous suspensions were frozen using liquid nitrogen and kept in the freezer overnight. Subsequently, the samples were freeze-dried for 24 h under ultra-high vacuum (0.05 mTorr) using Telstar LyoQuest-55 equipment.

### 2.5. Surface-Initiated ROP of LLA

All procedures were conducted under an inert atmosphere using standard Schlenk line techniques (O_2_ < 3 ppm) in conjunction with a MBraunUnilab-MB-20-G glove box (O_2_ < 0.6 ppm) (MBraun, Garching, Germany). All solvents were rigorously dried prior to use using the MBraun Solvent Purification System (MBraun, Garching, Germany). The [AlMeL_2_] complex was synthesized according to our previously reported procedure [20].

The polymerization reactions were performed under argon atmosphere in ampoules. Briefly, 17 mg of AlMeL_2_ was dissolved in dry toluene (2.0 mL) with the appropriate amount of monomer and CNCs (245.0 and 4.5 mg, respectively). Then, the reactions were conducted at 100 °C for 24 h in toluene. Aliquots from each reaction were taken and monitored by integrating the monomer and polymer signals in ^1^H-NMR spectra to evaluate the conversion. For comparison, the reference PLLA was synthetized under identical polymerization conditions using benzyl alcohol (BnOH) as a co-initiator in the absence of CNCs, at a molar ratio of 1:400 [BnOH]:[LLA].

### 2.6. Characterization Techniques

Nuclear Magnetic Resonance (NMR) spectra were recorded on a Bruker 400 Ultrashield (^1^H 400 MHz) at room temperature. Chemical shifts (δ) are reported in ppm and were referenced internally using CDCl_3_ as the solvent.

Thermogravimetric analyses (TGA) were carried out on a TGA55D analyzer (TA Instrument). Dynamic experiments were performed using about 10 mg of dried sample, which was heated from 25 to 600 °C at a rate of 10 °C/min under nitrogen atmosphere (60 mL/min). The maximum degradation rate (T_max_) was calculated from the first derivative of the TGA curves (dW/dT). The analysis was repeated for three different specimens of each freeze-dried sample to calculate the average values and their standard deviation.

Differential Scanning Calorimetry (DSC) analysis was carried out on a TA Instrument Discovery DSC25 (TA Instruments, New Castle, DE, USA), using a program consisting of heating/cooling/heating cycles from 0–200 °C with a heating/cooling rate of 10 °C/min under nitrogen purge (50 mL/min). Glass transition (T_g_), cold crystallization (T*_cc_*), and melting temperatures (T_m_) were measured from the second heating scan of the thermograms. The degree of crystallinity (X_c_) was calculated using Equation (1):

(1) $$\chi_{c}=100\times \left[\frac{{∆H}_{m}-{∆H}_{cc}}{{∆H}_{m}^{100}}\right]\times \left[\frac{1}{wf}\right]$$

where Δ*H_m_* is the enthalpy of fusion, Δ*H_cc_* is the enthalpy of cold crystallization, and Δ*H_m_*^100^ is the enthalpy of fusion of a 100 % crystalline PLA, taken as 93 J/g [27].

The morphology of the isolated cellulose was observed by scanning electron microscopy (SEM) using a Jeol JSM-IT500 instrument (JEOL Ltd., Tokyo, Japan) operated at 10 kV. Samples were sputter-coated with gold using a Polaron SEM coating system (1.4 kV, 1.8 mA, 120 min, thickness ≈ 500 Å) prior to imaging.

The samples were studied by transmission electron microscopy (TEM) using Jeol JEM2100HT equipment (JEOL Ltd., Tokyo, Japan) operated at 100 kV. One drop of the CNC dispersion in water was deposited on the grid with a carbon layer at room temperature until total solvent evaporation.

Attenuated total reflectance–Fourier transform infrared spectroscopy (ATR-FTIR) measurements were conducted using an Agilent Cary 630 FTIR Spectrometer (Agilent Technologies Inc., Santa Clara, CA, USA). Spectra were obtained in the 4000–400 cm^−1^ region at room temperature in transmission mode with a resolution of 4 cm^−1^.

A Bruker D8 Advance A25 diffractometer and Cu Kα radiation were used, while patterns were collected in steps of 0.02 (2θ between 5 and 40°) with a counting time of 1 s per step. The crystalline index of each sample was calculated using Equation (2):

(2) $$CI=100\times \frac{A_{crystal}}{A_{total}}$$

where A_crystal_ refers to the total area of the crystalline deconvoluted peaks and A_total_ refers to the total area of the diffractogram.

The analysis of the zeta potential was carried out using Malvern Nano ZS Zetasizer ZEN-3600 equipment (Malvern Panalytical Ltd., Malvern, UK). CNC diluted suspensions were prepared by sonication of each sample in 20 mL of deionized water in an ultrasound bath. All the measurements were conducted at neutral pH = 7 to assess surface charge and stability.

The molecular weights (M_n_ and M_w_) and dispersity values (Ɖ = M_w_/M_n_) of the polymers were determined by size-exclusion chromatography (SEC) on an Agilent 1260 Infinity II high-speed liquid chromatograph LC System equipped with two gradient columns PL gel 5 μm (Mixed-D and Mixed-C) connected in series. Tetrahydrofuran (THF) was the eluent, and the sample solutions were injected at a 1 mL·min^−1^ flow rate at 30 °C. The calibration was performed using polystyrene (PS) standards.

The mechanical properties of the samples were obtained from tensile stress–strain tests using an Instron 3345 universal testing system (Instron, Norwood, MA, USA) dotted with a 100 N load cell at room temperature. Rectangular specimens 20 mm in length, 5 mm in width, and 0.1 mm thick were tested, and the results from three specimens were averaged. The elastic modulus was obtained as the slope of the curve in the elastic region, while tensile strength and elongation were calculated in the break point.

## 3. Results and Discussion

### 3.1. Compositional Analysis of Orange Peel Waste

The compositional analysis of orange peel waste is summarized in Table 2. Based on glucose released during two-step acid hydrolysis, the cellulose content was estimated at 10.0 ± 1.0 g/100 g dry weight. Hemicellulose and acid insoluble residue contents were determined to be 7.0 ± 2.0 g/100 g and 3.6 ± 0.7 g/100 g, respectively. Although these fractions are not utilized in the CNC production process, their removal through the alkaline/peroxide treatment is essential. These values are comparable to those reported by Rivas et al., who described a composition of 9.2 g/100 g of cellulose, 10.5 g/100 g of hemicellulose, and 0.8 g/100 g of insoluble lignin in orange peel [28]. Compared to conventional lignocellulosic biomass—such as hardwoods, straws, or corn stover, which typically contain 30–50% of cellulose—the cellulose yield from orange peel waste is relatively low. However, citrus peel residues offer a distinct advantage: they are more amenable to chemical processing and require milder pretreatment conditions, making them attractive for sustainable and low-energy valorization pathways.

Moreover, water extraction removed a 52 ± 7% of the biomass dry weight, while ethanol extraction dissolved an additional 8 ± 1%. These fractions mainly correspond to soluble sugars, such as sucrose, glucose, and fructose [29], which has been reported to be up to 38–40% by weight [30], as well as organic acids, such as uronic acid [31,32], and other non-structural compounds of orange peel residues.

### 3.2. Morphological Characterization (SEM and TEM)

**Figure 1:**
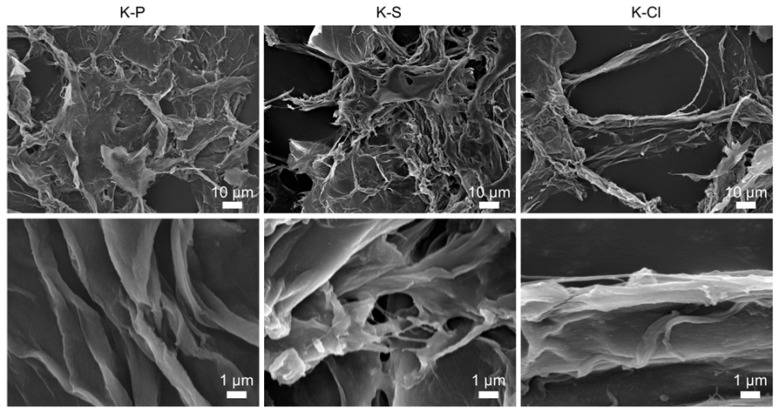
SEM images of alkaline/peroxide-treated orange waste after acid hydrolysis with H_3_PO_4_, H_3_SO_4_, and HCl at ×1000 and ×10,000 magnifications.

The morphology of cellulosic-based materials obtained after hydrolysis with different mineral acids was evaluated by scanning electron microscopy (SEM). Figure 1 shows the SEM images of samples treated with H_3_PO_4_ (K-P), H_2_SO_4_ (K-S), and HCl (K-Cl). All samples exhibited dense and compacted morphology. Although sample preparation for SEM was carefully carried out to avoid aggregation of cellulose fibers, avoiding thermal treatments, some aggregation was inevitable due to the possible hornification of fibrillated fibrils [33].

**Figure 2:**
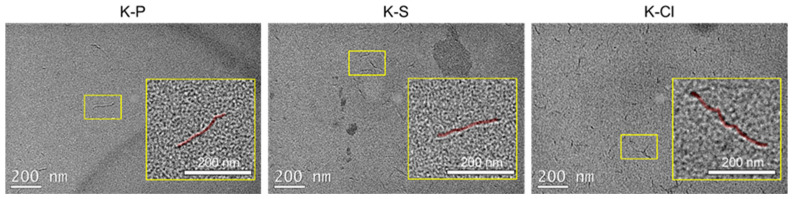
TEM images of alkaline/peroxide-treated orange waste after acid hydrolysis with H_3_PO_4_, H_3_SO_4_, and HCl.

Further structural details were revealed through transmission electron microscopy (TEM) because of its higher resolution (Figure 2). TEM imaging provided clearer evidence of nanofibrillation in all the samples, where individual CNCs were visible.

It is worth noting that all the samples showed CNCs with rod-like morphology. The CNCs observed showed average lengths of 229.3 nm, 236.5 nm, and 386.5 nm for K-P, K-S, and K-Cl, respectively. Based on the TEM observations, it can be concluded that the acid hydrolysis step was successfully achieved with the three acids used at diluted molar concentrations. The morphological changes observed in the samples provide clear evidence of effective degradation of the amorphous region on cellulose, indicating that the reaction conditions (time and temperature) were enough to promote cellulose breakage regardless of the acid used. Considering the diluted molar concentrations used, the lengths of the CNCs obtained are in the nanometric range, similar to those previously described in the literature. For instance, Camarero Espinosa et al. reported an average length of 316 ± 127 nm after acid hydrolysis with phosphoric acid (90 min, 100 °C, and 10.7 M) [13]. Kusmono et al. obtained needle-like shape CNCs with an average length of 145.61 nm under hydrolysis conditions (30 min, 45 °C, and 8.6 M) with sulfuric acid [34]. Finally, Araki et al. treated kraft pulp with HCl (225 min, 80 °C, and 4 M), obtaining CNCs with an average length of 180 ± 75 nm [26]. TEM analysis indicates that the overall morphology of the obtained CNCs remains consistent with that reported in previous studies. However, the use of significantly lower acid molar concentrations resulted in an increased average CNC length, demonstrating that higher acid conditions are required to promote more efficient hydrolysis and thus yield shorter CNCs.

### 3.3. FTIR Spectroscopy

**Figure 3:**
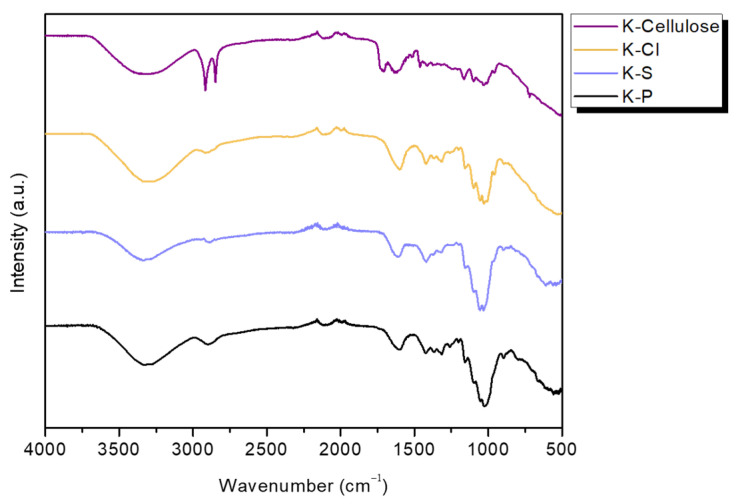
FTIR spectra for K-cellulose, K-P, K-S, and K-Cl.

FTIR spectroscopy was employed to assess the chemical structure of the hydrolyzed cellulose samples. Figure 3 shows the FTIR spectra of each sample, enabling comparison of their chemical structures and functional group profiles.

The spectra confirm the retention of characteristic cellulose functional groups across all the treatments, proving that the evaluated acid hydrolysis preserved the cellulose structure in all the samples. A broad absorption band near 3400 cm^−1^ is attributed to the stretching vibrations hydroxyl (-OH) groups, indicating the preservation of the cellulose backbone. A peak at 2900 cm^−1^ corresponds to -CH asymmetric stretching, while the band at 1429 cm^−1^ is assigned to the in-plane bending of -OCH groups. The peak at 1650 cm^−1^ is related to the -OH bending vibrations from absorbed water. Additionally, a band at 1372 cm^−1^ correlates with the -CH deformation in the cellulose, while the signal at 897 cm^−1^ band is attributed to the stretching vibrations of C-5 and C-6 atoms in the cellulose chemical structure [35]. Collectively, the FTIR results suggest that the cellulose structure was maintained following acid hydrolysis, regardless of the acid used. Regarding the FTIR spectra before (K-cellulose) and after (K-Cl, K-S, and K-P) the acid hydrolysis, some differences can be observed in the 3000–2800 cm^−1^ region. The hydrolyzed samples showed a broad peak at 2900 cm^−1^ which is usually ascribed to degraded cellulose [36]. However, for K-cellulose, two clear peaks can be observed at 2910 and 2896 cm^−1^ in the same region. The two latter bands are clearly resolved in the FTIR spectra and attributed to the asymmetric and symmetric stretching vibration of aliphatic -CH groups in cellulose [36]. The changes observed in the number and intensity of the FTIR bands suggest alterations in the internal structure and crystalline order of the samples. These modifications are attributed to acid hydrolysis, which primarily targets the amorphous regions of cellulose. Therefore, the crystallinity of the samples will be examined and widely described in the following section.

Several studies have established a correlation between the FTIR spectra and the crystallinity of cellulose materials. Specific peak ratios can be used to estimate different aspects of crystallinity and molecular order. In particular, the IR crystallinity ratio is the ratio of a peak representing crystallinity to a peak not representing crystallinity within the same spectrum. The lateral order index (LOI), described by Hurtubise and Krassig [37], is defined as the ratio between the absorbance at 1429 cm^−1^, associated with the amount of crystalline structure of cellulose, and that at 897 cm^−1^, associated with the amorphous region in cellulose, reflecting the degree of structural order [38]. Moreover, the total crystallinity index (TCI) is calculated as the ratio of absorbance at 1372 cm^−1^ (C-H bending in crystalline cellulose) to 2900 cm^−1^ (C-H stretching), as described by Nelson and O’Connor [39]. While the TCI is proportional to the crystallinity degree of cellulose, the LOI is correlated to the overall degree of order in cellulose. Together, these indices offer a multi-faceted view of the structural organization within cellulose. The TCI and LOI values calculated for the K-cellulose and acid-treated samples are summarized in Table 3.

As shown in Table 3, the TCI increased significantly in all acid-treated samples (0.79–0.86) compared to the K-cellulose (TCI = 0.50), indicating the successful removal of amorphous regions. These results evidence that all three inorganic acids, even at relatively low molar concentrations (3.0–4.5 mol/L), can be used to obtain cellulose-based materials with high crystallinity derived from orange peel waste.

Among the treatments, the sulfuric acid-treated sample (K-S) achieved the highest TCI value (0.86), suggesting that sulfuric acid treatment (3.0 mol/L, 80 °C, 225 min) was the most effective at producing high-crystalline cellulose. Interestingly, the phosphoric acid treatment (4.5 mol/L, 100 °C, 90 min) achieved a similar TCI value (0.81) under shorter reaction times (90 min). Finally, the lowest TCI value (0.79) was obtained using the hydrochloric acid treatment (3.6 mol/L, 45 °C, 60 min), likely due to its relatively mild conditions, which may have been insufficient for extensive amorphous cellulose removal. It is worth noting that all the TCI values were higher than those reported for other cellulose nanofibers obtained from other natural sources. For instance, Poletto et al. reported a TCI value of 0.457 ± 0.02 for cellulose nanofibers *Eucalyptus grandis* and a TCI value of 0.491 ± 0.01 for *Pinus taeda*, both obtained by a sulfite pulping process of kraft [40].

It is important to highlight that while both TCI and LOI are indicators of cellulose crystallinity, they assess different aspects of the structure. TCI reflects the overall degree of crystallinity, while LOI is more sensitive to the ordered arrangement of cellulose chains within the crystalline regions. From this perspective, the K-S sample showed the highest LOI value (1.51), which reveals a more ordered and tightly packed crystalline structure in cellulose, probably due to the presence of sulfate groups. However, this behavior was not observed in the K-P sample, for which the LOI value was similar to that of the untreated K-cellulose sample (0.88 and 0.85 respectively). Given the complex chemical composition of the raw material and the different reaction conditions used with each acid, it is difficult to draw more definitive conclusions regarding the internal crystalline organization of the cellulosic products.

To draw more conclusive insights into the internal crystalline organization, X-ray diffraction analysis was performed, which provides further details about the crystallographic planes (see Supporting Information). The XRD patterns display characteristic crystalline peaks of cellulose at 2θ = 15 and 22°, corresponding to the [110] and [200] crystal planes, respectively [34]. The crystallinity index of each sample (see Table 3) followed the same trend previously observed in the FTIR analysis. The highest value was observed for K-S (35.2%), followed by K-P (28.6%), whereas K-Cl exhibited the lowest value (26.0%). These findings confirm that the processing conditions directly influence the internal crystalline organization, removing the amorphous part during the acid hydrolysis step.

### 3.4. Thermal Degradation Behavior

**Figure 4:**
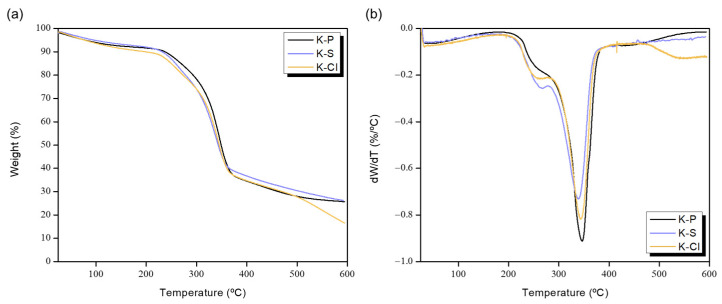
(**a**) TGA curves showing the weight loss in the K-P, K-S, and K-Cl samples; (**b**) TGA derivatives curves for K-P, K-S and K-Cl samples.

To assess the thermal stability of the cellulose products obtained after the acid hydrolysis of alkaline/peroxide-treated orange peel waste, thermogravimetric curves and their derivatives (dW/dT) are presented in Figure 4 for the different samples. Moreover, the average thermogravimetric curves and data of the different K-cellulose samples after acid hydrolysis (K-S, K-P, and K-Cl) are reported with their standard deviations in the Supporting Information. Each peak observed in Figure 4b is attributed to a distinct degradation stage of the material, as detailed in Table 4. All the samples exhibited initial weight loss, corresponding to the evaporation of water at 100 °C. Thermal degradation occurred over a wide temperature range and showed a complex decomposition profile. It has been previously suggested that highly sulfated samples degrade through a two-step process [41]. In our study, all the samples showed an initial degradation step with onset at 200 °C, and a maximum weight-loss rate around 260 °C. In general, during the acid hydrolysis of the cellulose, the acid protons rapidly protonate the glycosidic oxygen, followed by slow cleavage of glycosidic bonds induced by the addition of water [42]. For this reason, the amorphous region hydrolyzes quickly, leaving the crystalline regions intact in the acid aqueous solution. In our case, the hydrolysis of cellulose was performed with mineral acids. The resulting hydroxyl group can undergo partial esterification with the sulfate or phosphate species [41]. These modifications influence both the surface charge and the thermal stability of cellulose. The prominent initial thermal degradation step at 260 °C corresponds to the decomposition of highly sulfated and phosphate regions. This fact is further supported by the final residual weight observed in K-S and K-P (26.2 and 25.7%, respectively), consistent with the incorporation of sulfate and phosphate groups.

In contrast, cellulose treated with HCl acid (K-Cl sample) is not expected to undergo such esterification, as HCl does not introduce charged inorganic groups. Typically, HCl-treated cellulose degrades faster during the first step, showing a higher weight loss at this temperature. However, almost the same weight loss was observed for all the samples in our study (13.1, 14.9 and 14.0% for K-P, K-S and K-Cl, respectively). This early weight loss is often attributed to the decomposition of residual hemicellulose (amorphous structure) [42]. This result supports that the K-Cl sample retained a higher amount of amorphous fraction, likely a result of the milder hydrolysis conditions employed, an observation further corroborated by the FTIR and crystallinity analyses.

The second degradation step corresponds to the final depolymerization of the crystalline cellulose. All the samples showed a T_m_ around 340 °C, which is consistent with the reported thermal stability of crystalline cellulose [41,43,44].

Finally, from Table 4 it can also be seen that there were differences in final residue among all CNCs. This is probably attributed to differences in the chemical structure (sulfate or phosphate groups) and crystallinity of CNCs. K-P and K-S showed similar values of char residue, while K-Cl showed the lowest value. The final residues of CNCs decreased according to the crystallinity index (Table 3). These results were found to be similar to the results previously reported [34,45,46].

### 3.5. Zeta Potential Analysis

The surface charge of each sample after hydrolysis was studied by zeta potential analysis, and the results are summarized in Table 5. Zeta potential is a measure of the electrical charge at the interface between a particle and its surrounding solvent, which can be directly correlated to the aggregation trend of CNCs. Hydrolysis with sulfuric and phosphoric acids partially esterifies the surface hydroxyl groups with sulfate or phosphate groups, increasing the negative surface charge of CNCs [8]. The presence of these sulfate and phosphate groups usually increases stabilization due to mutual repulsion preventing the aggregation of CNCs. In contrast, CNCs obtained with hydrochloric acid usually tend to flocculate because of the lower surface charge due to the lack of inorganic anionic groups [8].

Among the samples, the highest zeta potential value was obtained for K-S (−28.8 ± 1.7 mV), confirming the presence of sulfate and hydrogen sulfate groups. In fact, Aguayo et al. confirmed that the sulfur content in CNCs is directly correlated with the zeta potential value [47]. Based on their results, an 11.6 mg/g sulfur content can be approximately estimated in our samples considering the zeta potential value. Moreover, K-P showed a value of −27.5 ± 1.9 mV, which is in the range of previously reported values for similar phosphorylated CNCs [48], supporting the incorporation of phosphate groups. Conversely, the treatment with hydrochloric acid displayed the lowest zeta potential value of −26.2 ± 1.0 mV, which aligns with previously reported zeta potential values for similar CNCs obtained with HCl treatment [23]. It is worth noting that the zeta potential is strongly influenced by pH. In this study, all measurements were conducted at neutral pH (7.0) to ensure comparability across samples.

### 3.6. Surface-Initiated ROP of Lactide

**Figure 5:**
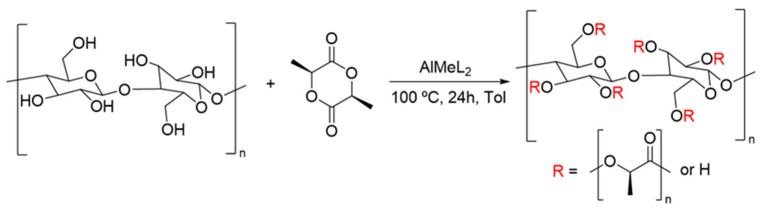
Schematic reaction of the SI-ROP of LLA organocatalyzed by the AlMeL_2_ complex.

Based on the results obtained, the surface-initiated ring-opening polymerization of *L*-lactide was carried out using freeze-dried samples (K-P, K-S, and K-Cl), as reported in Figure 5.

The polymerization reactions were conducted with a fixed concentration of [AlMeL_2_] (0.015 mol/L) and a *L*-LA-to-cellulose ratio of 50:1, as summarized in Table 6.

**Figure 6:**
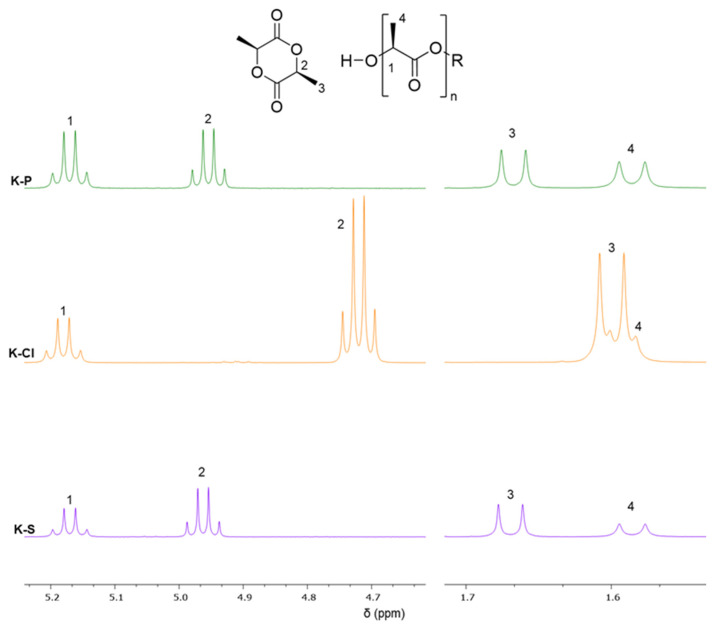
^1^H-NMR spectra (CDCl_3_) of aliquots from reactions of entries 1, 2, and 3 in Table 6, 4 is related to CH_3_ protons in the PLLA chains.

The polymerization progress was monitored using ^1^H-NMR, taking an aliquot from the reaction mixture. The NMR spectra are displayed in Figure 6, showing the characteristic signals in the regions of 5.10–5.20 ppm and 4.90–5.00 ppm. The quadruplets at higher chemical shifts correspond to C-H protons in the PLLA chains (5.10–5.20 ppm), while those for the C-H protons of the unreacted monomer *L*-LA appear at lower shift values (4.90–5.00 ppm). Opposite behavior was observed for the methyl protons that appeared at 1.50 and 1.70 ppm, respectively. The presence of both quadruplets is due to the fact that no complete conversion was achieved in the reaction time studied (24 h). While ^1^H-NMR confirmed the formation of PLLA, its utility was limited in elucidating the grafting efficiency or CNC–polymer interface due to the high LLA/CNC ratio and the solid-state nature of the initiators.

The presence of the characteristic signals corresponding to polymerized lactide in all ^1^H-NMR spectra confirm the catalytic activity of the [AlMeL_2_] complex in the presence of K-P, K-S, or K-Cl as co-initiators. The calculated monomer conversions were 55, 43, and 24% for K-P, K-S, and K-Cl, respectively. It is worth noting that the [AlMeL_2_] complex has previously demonstrated high efficiency under rigorously anhydrous conditions. In contrast, the CNCs used in this study are highly hydrophilic and retain residual moisture even after freeze-drying, which may have partially compromised the catalytic activity of the [AlMeL_2_] complex. Interestingly, in the K-Cl sample, the signals attributed to methyl protons (1.50–1.70 ppm) appear overlapped, and the quadruplet in the higher field appears shifted. This behavior may be consistent with the presence of low-molecular-weight oligomers produced during the SI-ROP. Protons of short oligomers next to chain-end groups can overlap in the ^1^H-NMR spectra [23].

Following polymerization, the crude reaction mixtures were purified and isolated by precipitation in EtOH and vacuum-dried at 60 °C. The purified polymers were then analyzed by SEC to determine their molecular weights (Table 6). It is important to note that polymer isolation from the K-Cl sample was particularly difficult, likely due to its low molecular weight and poor precipitation behavior. Nevertheless, a small quantity of product (mg) was recovered and characterized.

Furthermore, for reference, PLLA was synthetized under identical polymerization conditions, using benzyl alcohol (BnOH) as a co-initiator in the absence of CNCs (PLLA 60 kDa), following the methodology previously described by us [21]. In this case, the molecular weight achieved was M_w_ = 60 kDa and Đ = 1.66. The mechanism for this ROP polymerization involves the role of BnOH as the co-initiator, as has been reported by us (see Supporting Information).

All samples (except for PLLA 60 kDa) exhibited bimodal molecular weight distribution in the SEC chromatograms (see Supporting Information). This double peak might be attributed to incomplete or non-uniform dispersion of the CNC initiators in the reaction media. If the number of initiators is lower relative to the LLA monomer ratio, there will be fewer initiating chains, which will result in longer polymer chains. Conversely, improved dispersion leads to a higher number of accessible initiation sites, yielding shorter polymer chains. However, from a mechanistic point of view, the presence of chains with two different molecular weights could also be related to the presence of competing mechanisms that may involve different initiation pathways. As such, in the case of K-P and K-S, the presence of oxoanions in the residues, such as sulfate, hydrogensulfate, phosphate, and hydrophosphate, as detected in the zeta potential measurements, could act as alternative initiators competing with the OH groups already present in the CNC surfaces. This alternative initiator would not occur for K-Cl, which is in agreement with the different behavior observed for this sample.

Despite these uncertainties, the molecular weight of PLLA grafted onto CNCs in this study were higher than those previously reported for similar systems. For example, Lalanne-Tisné et al. employed DMAP as an organocatalyst to polymerize lactide from cellulose nanofibers, obtaining average molecular weights between 600 and 1400 g/mol [23].

**Figure 7:**
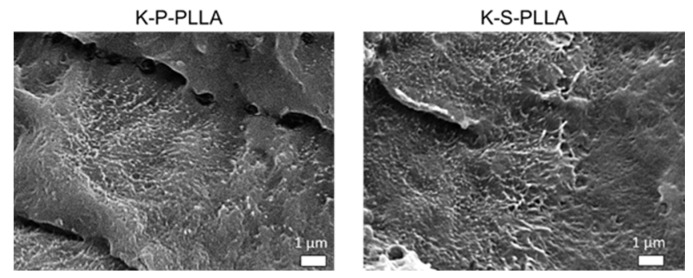
SEM images of fracture surfaces of K-P-PLLA and K-S-PLLA samples.

The isolated PLLA-based composites obtained were dissolved and used to prepare films by the solvent-casting technique in order to study its fracture surface by SEM (see Figure 7). Unfortunately, it was impossible to obtain a film for SEM analysis for the K-Cl-PLLA composite, probably due to the fact that there were oligomers, since the isolated fraction with high molecular weight was negligible.

On the SEM images of the fracture surfaces of each composite, a homogeneous distribution of CNCs throughout the polymer matrix can be observed. Notably, both samples exhibited a good interfacial adhesion between the PLLA and the fillers, likely resulting from the grafting of the LLA onto the CNC surface. It is important to highlight the slight agglomeration of CNCs in the K-S-PLLA sample, which is probably due to the higher tendency of the sulfate groups to be involved in stronger interactions, increasing the tendency for agglomerations in this formulation.

The thermal properties of the purified samples were subsequently characterized, and the results are summarized in Table 7.

**Figure 8:**
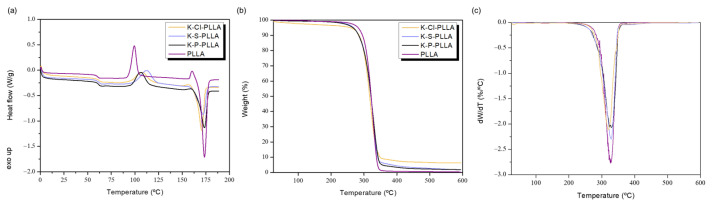
(**a**) Second heating scan thermograms for each sample at 10 °C/min; (**b**) TGA curves showing the weight loss of each sample; (**c**) TGA derivative curves for each sample.

The amount of PLLA grafted to the K-P, K-S, and K-Cl samples was calculated from the TGA curves (see Figure 8b). In all cases, the amount of PLLA grafted was higher than 90%, with the lowest value observed for the K-Cl-PLLA sample. This aligns with its lower monomer conversion, as determined by ^1^H-NMR. Notably, all TGA curves exhibited a broad shoulder overlapping with the main degradation peak, which can be attributed to the second thermal degradation step previously described for CNCs.

Differential Scanning Calorimetry (DSC) analysis (Figure 8a), performed using the second heating scan, revealed a consistent glass transition temperature (T_g_) of 61 °C across all samples, and melting temperatures (T_m_) in the range of 171–174 °C. These values are consistent with those reported for isotactic PLLA [49]. However, the presence of CNCs derived from different acid hydrolysis appears to have influenced the semicrystalline nature of the PLLA. When compared with PLLA obtained using the same catalyst and benzyl alcohol (BnOH), the degree of crystallinity of PLLA strongly decreases, reaching the minimum value for K-S-PLLA. This behavior can be due to the fact that the CNCs attached to the PLLA chains can hinder the chain reorganization for crystallizing, leading to a decrease in the PLLA chain mobility. Similar results were reported for PLA/PCL blends filled with functionalized CNCs [27].

**Figure 9:**
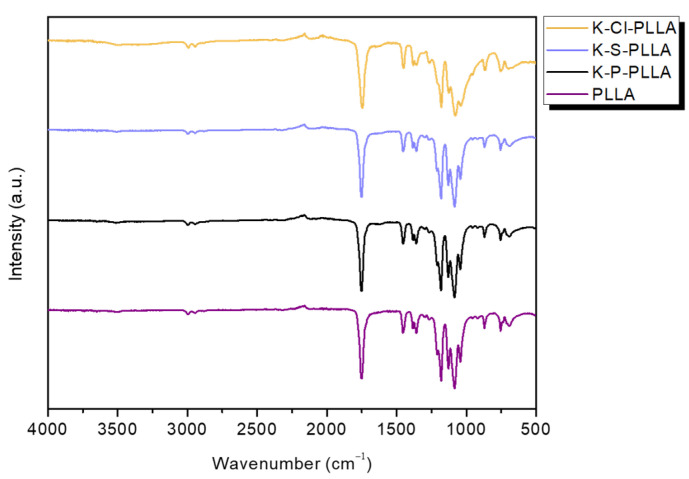
FTIR spectra for the isolated polymers obtained from reactions of entries 1, 2, and 3 in Table 6 and a PLLA spectra for comparison.

The FTIR spectra of all the samples have been compared with a PLLA synthetized with the same conditions in the absence of cellulose (Figure 9).

FTIR spectra confirmed the presence of the characteristic PLLA functional groups in all samples. The prominent band at 1750 cm^−1^ is assigned to the stretching band of the carbonyl group [50]. Both the asymmetric and symmetric deformation modes of CH_3_ at 1450 and 1386 cm^−1^, respectively, were observed in all the samples. Moreover, the two bands at 2995 and 2940 cm^−1^ are associated with the asymmetric and symmetric stretching of CH_3_ [50]. Finally, the band at 1080 cm^−1^ is correlated with the C-O asymmetric mode, and it was observable in all the samples [50].

These results indicate that the PLLA chains obtained by SI-ROP from CNCs had the same chemical structure as PLLA synthesized in the absence of cellulose. Due to the low amount of CNCs in the samples, the typical cellulose peaks were not clearly observable in the FTIR spectra. However, in the K-Cl-PLLA spectra, a slight broad peak in the region of 3500 cm^−1^ was detected, which may be attributed to the presence of residual unreacted hydroxyl groups in CNCs. This observation is consistent with the lower conversion rate for this sample.

Finally, the mechanical properties of neat PLLA and the different composites reinforced with CNCs were evaluated and are summarized in Table 8. While cellulose nanocrystals are generally expected to enhance stiffness and strength at a relatively low concentration (1 wt%), in this case, the relatively high CNC loading obtained (6 and 7 wt% for K-P-PLLA and K-S-PLLA, respectively) resulted in a loss of the reinforcing effect. The elastic modulus decreased slightly from 886.5 MPa in neat PLLA to 784.7 and 537.2 MPa in the composites, and the maximum tensile stress dropped accordingly from 16.1 to 9.5 and 9.6 MPa. However, a slight increase in elongation at break was observed for K-P-PLLA. These results suggest that the mechanical properties of PLLA-CNC composites could be tailored by carefully adjusting the proportions used during the synthesis. Note that the main focus of this work is not to obtain the best performing composites in terms of mechanical properties, but rather to demonstrate that the incorporation of CNCs has considerable influence on the mechanical performance of the obtained materials.

## 4. Conclusions

This study demonstrates the revalorization of orange peel waste as a source of cellulose nanocrystals (CNCs) and their subsequent application as co-initiators in the ring-opening polymerization (ROP) of *L*-lactide to produce polylactide (PLLA).

Following an alkaline/peroxide treatment, CNCs were successfully isolated using mild acid hydrolysis with inorganic acids such as sulfuric, phosphoric, and hydrochloric acid. The resulting materials exhibited distinct structural and physicochemical properties depending on the acid, with sulfuric acid treatment (K-S) yielding the highest crystallinity index (TCI = 0.86) even at a low concentration (3.0 mol/L).

Thermal analysis revealed that CNCs modified with sulfate or phosphate groups (K-S and K-P) exhibited increased thermal stability and higher residual mass, indicating successful surface functionalization. The zeta potential values (−26.2 to −28.8 mV) further support this behavior, as more negative surface charges for K-S and K-P compared to K-Cl were observed, in agreement with the aggregation behavior detected in TEM and the presence of anionic functional groups. These results suggest that the choice of inorganic acid can be used to tailor the final properties of CNCs for specific applications.

The CNCs were employed as solid co-initiators for the [AlMeL_2_]-catalyzed SI-ROP of *L*-lactide. The conversion and molecular weight of the resulting PLLA varied with the type of CNCs used and its dispersion in the solution. The polymers obtained were thermally characterized, showing a glass transition temperature value similar to commercial PLLAs, as well as a melting temperature value in the range of 171–174 °C. The amount of PLLA grafted onto the CNCs was 90, 93, and 94% for the CNCs treated with hydrochloric, sulfuric, and phosphoric acid, respectively.

Overall, this work highlights the dual role of CNCs derived from agro-industrial waste as both sustainable materials and functional co-initiators for PLLA synthesis. Future studies should aim to further elucidate the grafting mechanism using solid-state NMR spectroscopy and optimize the dispersion and surface functionalization of CNCs to enhance their catalytic performance in SI-ROP reactions.

## Figures and Tables

**Table 1:** Conditions used for acid hydrolysis.

Code	Acid	M (mol/L)	Temp. (°C)	Time (min)	Reference
K-P	H_3_PO_4_	4.5	100	90	Adapted from [13]
K-S	H_2_SO_4_	3.0	80	225	Adapted from [25]
K-Cl	HCl	3.6	45	60	Adapted from [26]

**Table 2:** Extractives (water and ethanol), sugar, and acid insoluble residue (lignin) determined for orange bagasse.

Compounds (g/100 g Dry Weight)		
Extractives	Water	52 ± 7
	Ethanol	8 ± 1
Sugars in water extractives	Sucrose	Nd
	Glucose	9.8 ± 0.9
	Fructose	9.9 ± 0.5
	Xylose	0.3 ± 0.0
	Galactose	0.14 ± 0.0
	Arabinose	<0.1
	Mannose	<0.1
Glucan	Glucose	10 ± 1
Hemicellulose (7.9%)	Xylose	1.5 ± 0.04
	Galactose	4.1 ± 0.5
	Arabinose	4.6 ± 0.3
	Mannose	1 ± 0.3
Lignin	Acid-insoluble residue	1.9 ± 0.4
	Acid-soluble residue	1.7 ± 0.3
Ash		3.81 ± 0.0

**Table 3:** Cellulose infrared crystallinity ratios and crystallinity index for each sample.

Code	IR Crystallinity Ratio		Crystallinity Index (%)
	1372 cm^−1^/2900 cm^−1^ (TCI)	1429 cm^−1^/897 cm^−1^ (LOI)	(A_cryst_/A_total_) · 100
K-Cellulose	0.50	0.85	-
K-P	0.81	0.88	28.6
K-S	0.86	1.51	35.2
K-Cl	0.79	1.00	26.0

**Table 4:** Thermal properties obtained from TGA curves for each sample.

Sample	T_max1_ (°C)	Weight Loss (%)	T_max2_ (°C)	Weight Loss (%)	Char Residue at 500 °C (%)
K-P	259	13.1	346	42.1	25.7
K-S	262	14.9	339	38.0	26.2
K-Cl	257	14.0	345	41.2	16.5

**Table 5:** Average zeta potential values for K-P, K-S, and K-Cl samples.

Sample	Zeta Potential (mV)
K-P	−27.5 ± 1.9
K-S	−28.8 ± 1.7
K-Cl	−26.2 ± 1.0

**Table 6:** SI-ROP reactions conducted in toluene at 100 °C and 24 h and molecular weights obtained by SEC.

Entry	Sample	LLA/Cellulose Weight Ratio	[LLA] M (mol/L)	Conv. ^a^ (%)	M_w, exp_ ^b^ (kDa)	M_n, exp_ ^b^ (kDa)	Đ ^b^
1	K-P	50:1	0.85	55	203.3	185.3	1.09
					67.1	48.8	1.07
2	K-S	50:1	0.85	43	144.9	134.5	1.07
					52.5	48.8	1.07
3	K-Cl	50:1	0.85	24	65.0	55.4	1.10
					14.6	13.3	1.09

^a^ Determined by ^1^H-NMR spectroscopy. ^b^ Determined by SEC in THF, using polystyrene standards. [AlMeL_2_] concentration: 0.015 mol/L.

**Table 7:** Thermal properties obtained from DSC and TGA for each sample.

	2nd Heating				TGA	
Sample	T_g_ (°C)	T_cc_ (°C)	T_m_ (°C)	X_c_ (%)	T_max_ (°C)	Grafted PLLA (wt%)
PLLA (60 kDa)	60	99	173	17.2	326	-
K-P-PLLA	61	107	174	5.7	328	94
K-S-PLLA	61	113	172	3.8	327	93
K-Cl-PLLA	61	108	171	10.6	320	90

**Table 8:** Mechanical properties for each sample.

Sample	E (MPa)	σ (MPa)	ε_break_ (%)
PLLA (60 kDa)	886.5 ± 7.6	16.1 ± 3.6	1.9 ± 0.5
K-P-PLLA	784.7 ± 107.1	9.5 ± 5.0	2.4 ± 0.2
K-S-PLLA	537.2 ± 80.9	9.6 ± 2.9	1.5 ± 1.3

## Data Availability

The original contributions presented in the study are included in the article/Supplementary Material, further inquiries can be directed to the corresponding authors.

## Supplementary Materials

The following Supporting Information can be downloaded at https://www.mdpi.com/article/10.3390/polym17192605/s1: Figure S1. XRD diffractogram of K-P; Figure S2. XRD diffractogram of K-S; Figure S3. XRD diffractogram of K-Cl; Figure S4. Scheme of polymerization mechanism of *L*-lactide catalyzed by AlMeL_2_ in presence of BnOH as co-initiator; Figure S5. Complete ^1^H-NMR spectra (CDCl_3_) of aliquots from reactions of entries 1, 2, and 3 in Table 6; Figure S6. SEC chromatogram for PLLA; Figure S7. SEC chromatogram for K-P-PLLA; Figure S8. SEC chromatogram for K-S-PLLA; Figure S9. SEC chromatogram for K-Cl-PLLA. Figure S10. TGA and derivatives curve for PLLA sample. Figure S11. Stress—strain tensile curves for each sample; Figure S12. Average TGA curve with standard deviation for K-S (obtained with 3 specimens); Figure S13. Average TGA curve with standard deviation for K-P (obtained with 3 specimens); Figure S14. Average TGA curve with standard deviation for K-Cl (obtained with 3 specimens); Table S1. Thermal properties obtained from average TGA curves for each sample (obtained with 3 freeze-dried specimens).
