# Supplementary material for: Upcycling Orange-Based Waste into Functional CNCs for Greener L-Lactide Ring-Opening Polymerization
Source: Polymers (Basel). 2025 Sep 26;17(19):2605. doi: 10.3390/polym17192605 (PMC12526882; doi:10.3390/polym17192605)
Supplement: Supplementary file 1 [file polymers-17-02605-s001.zip › polymers-3820522-supplementary.pdf]

Supporting Information

Upcycling Orange-Based Waste into Functional CNCs for Greener *L*-Lactide Ring-Opening Polymerization

Adrián Leonés [1,*], Cayetano Sánchez-Solís [1], Asier Medel [1], Maria P. García-Aparicio [2], Marta E. G. Mosquera [1,*] and Valentina Sessini [1,*]

[1] Departamento de Química Orgánica y Química Inorgánica, Facultad de Ciencias, Universidad de Alcalá, Ctra. Madrid-Barcelona Km. 33.6, Madrid, 28805 Alcalá de Henares, Spain; cayetano.sanchez@edu.uah.es (C.S.-S.); asier.medel@uah.es (A.M.)
[2] Advanced Biofuels and Bioproducts Unit, Renewable Energy Division, CIEMAT, 28040 Madrid, Spain; m.delprado@ciemat.es
* Correspondence: adrian.leones@uah.es (A.L.); martaeg.mosquera@uah.es (M.E.G.M.); valentina.sessini@uah.es (V.S.)

Index

1. XRD diffractograms
2. Polymerization mechanism
3. ^{1}H-NMR spectra
4. Size-exclusion chromatographic results
5. Thermogravimetric analysis of PLLA reference sample
6. Stress–strain tensile curves
7. Thermogravimetric analysis of K-cellulose after acid hydrolysis

1. XRD diffractograms

K-P

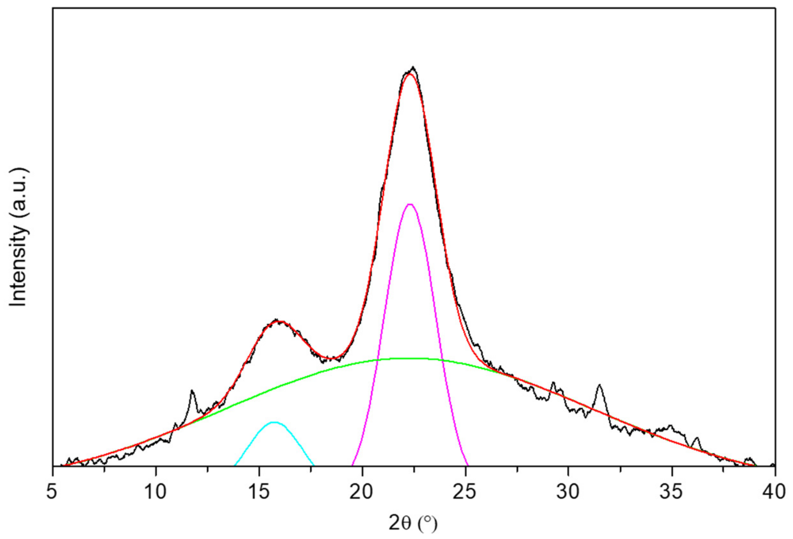

Figure S1. XRD diffractogram of K-P.

Peak	Peak Type	FWHM	Max. Height	Center Grvty	Area Intg
Peak 1	Gaussian	3.10758	181.03726	15.74927	598.85683
Peak 2	Gaussian	2.94875	776.30188	22.31433	2436.69262
Amorphous	Gaussian	21.09335	355.7641	22.25295	7582.7868

K-S

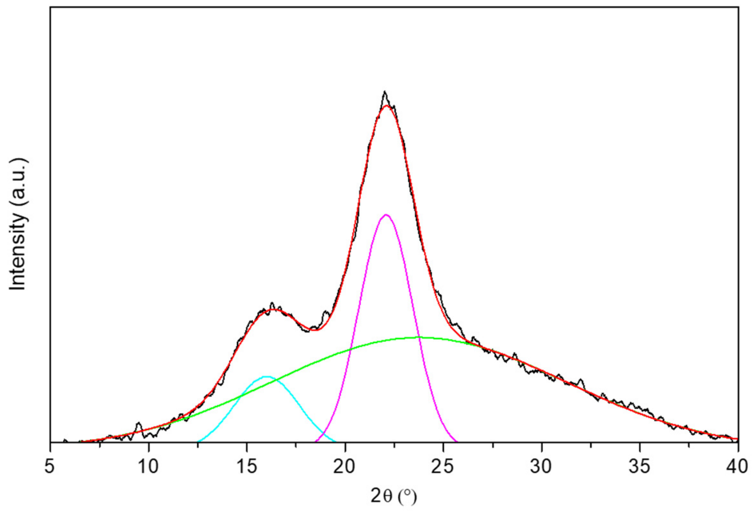

Figure S2. XRD diffractogram of K-S.

Peak	Peak Type	FWHM	Max. Height	Center Grvty	Area Intg
Peak 1	Gaussian	3.9165	252.02088	16.01309	1050.67184
Peak 2	Gaussian	3.24678	808.35538	22.07623	2793.74504
Amorphous	Gaussian	17.58874	386.7542	23.75521	7090.78001

K-Cl

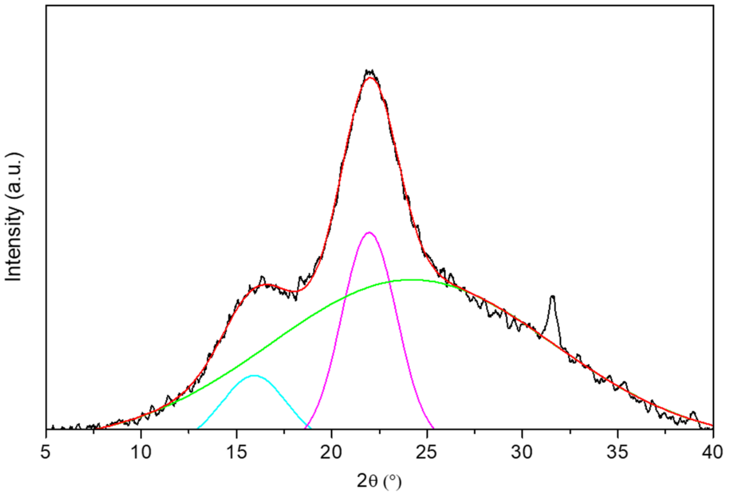

Figure S3. XRD diffractogram of K-Cl.

Peak	Peak Type	FWHM	Max. Height	Center Grvty	Area Intg
Peak 1	Gaussian	3.9521	126.04654	15.93141	530.26222
Peak 2	Gaussian	3.40361	395.84337	21.95986	1434.15121
Amorphous	Gaussian	17.44562	307.10884	24.19618	5582.50347

2. Polymerization mechanism

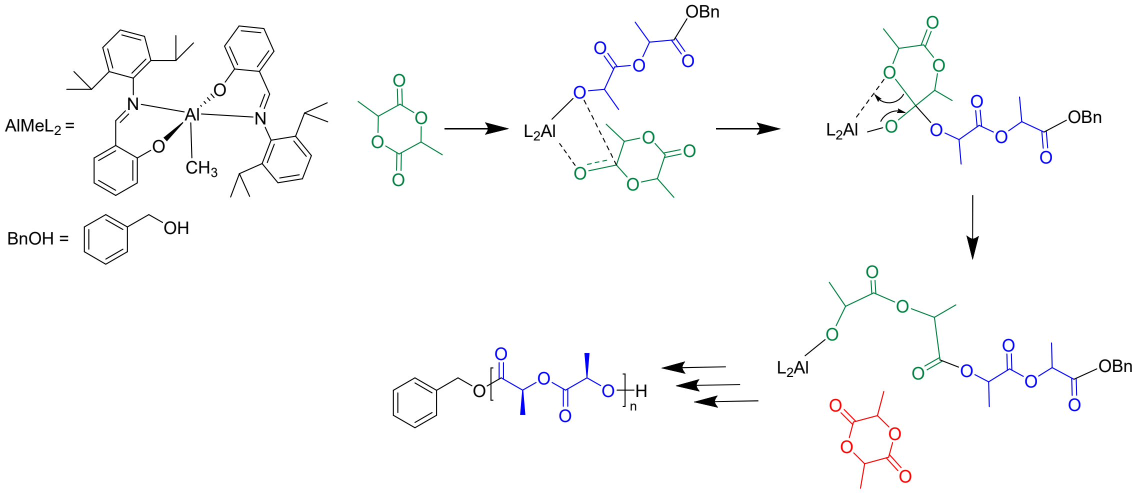

Figure S4. Scheme of polymerization mechanism of *L*-lactide catalyzed by $AlMeL_2$ in presence of BnOH as co-initiator.

3. ^{1}H-NMR spectra

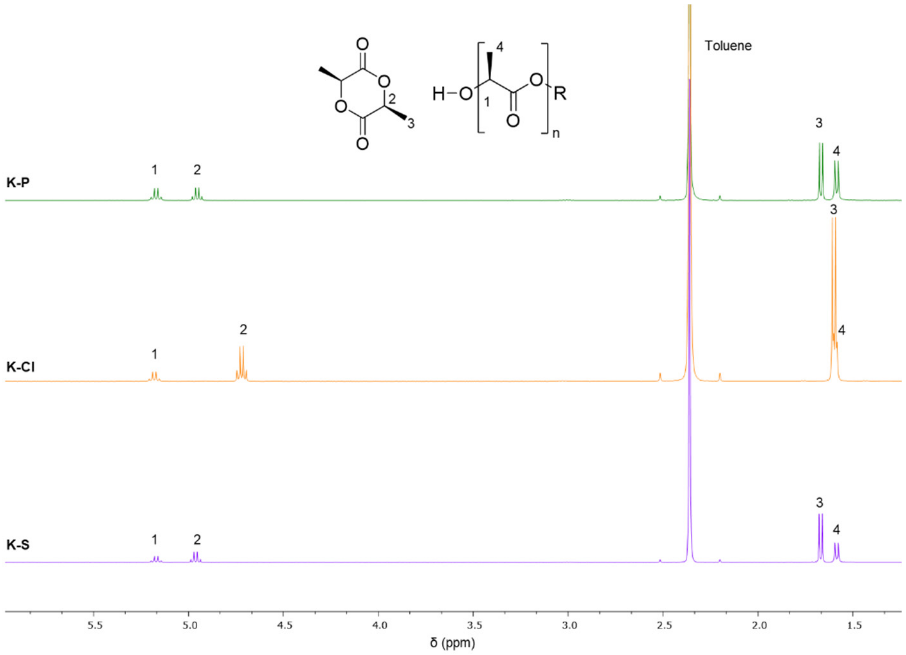

Figure S5. Magnifications of the ^{1}H-NMR spectra ($CDCl_3$) of aliquots from reactions of entries 1, 2, and 3 in Table 6.

4. Size-Exclusion Chromatographic results

The molecular weight values of the different polymers were obtained using a 10-point calibration curve based on PS standards.

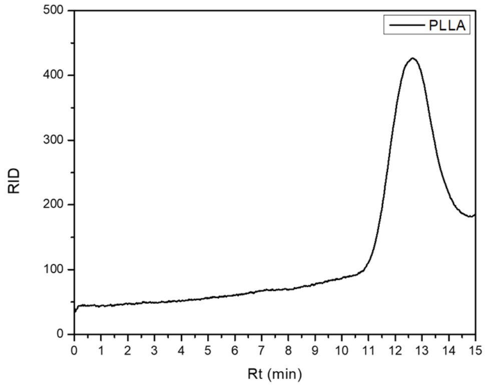

Figure S6. SEC chromatogram for PLLA.

Peak	Start (min)	End (min)	M_n (kDa)	M_w (kDa)	Đ
Peak 1	10.88	14.46	36.0	60.0	1.66

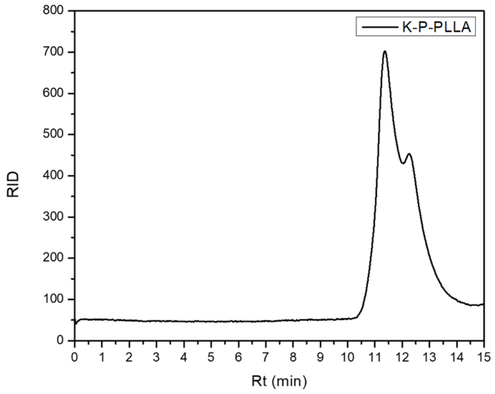

Figure S7. SEC chromatogram for K-P-PLLA.

Peak	Start (min)	End (min)	M_n (kDa)	M_w (kDa)	Đ
Peak 1	10.27	11.91	185.3	203.3	1.09
Peak 2	11.91	13.47	48.8	67.1	1.07

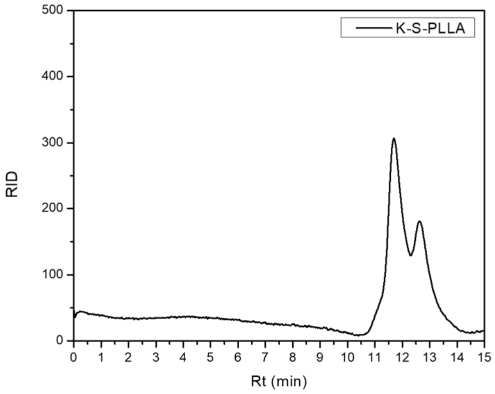

Figure S8. SEC chromatogram for K-S-PLLA.

Peak	Start (min)	End (min)	M_n (kDa)	M_w (kDa)	Đ
Peak 1	10.60	12.20	134.5	144.9	1.07
Peak 2	12.20	13.37	48.8	52.5	1.07

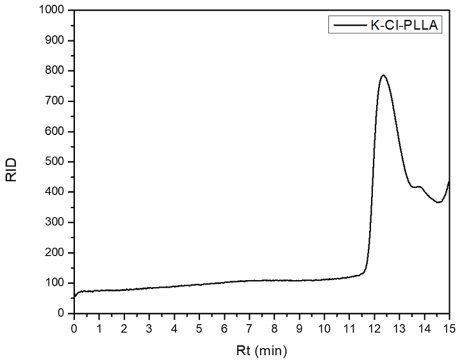

Figure S9. SEC chromatogram for K-Cl-PLLA.

Peak	Start (min)	End (min)	M_n (kDa)	M_w (kDa)	Đ
Peak 1	11.41	13.39	55.4	65.0	1.10
Peak 2	13.39	14.62	13.3	14.6	1.09

5. Thermogravimetric analysis of PLLA reference sample

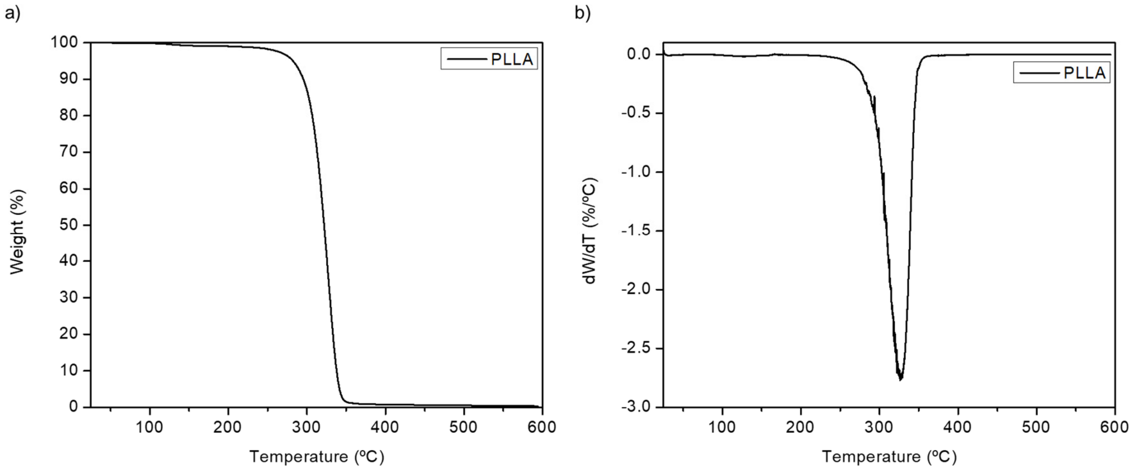

Figure S10. TGA and derivatives curves for PLLA sample.

6. Stress–strain tensile curves

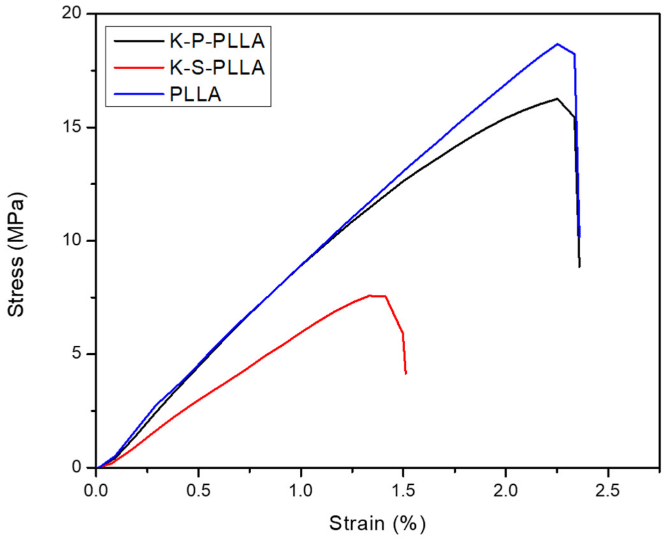

Figure S11. Stress–strain tensile curves for each sample.

7. Thermogravimetric analysis of K-cellulose after acid hydrolysis

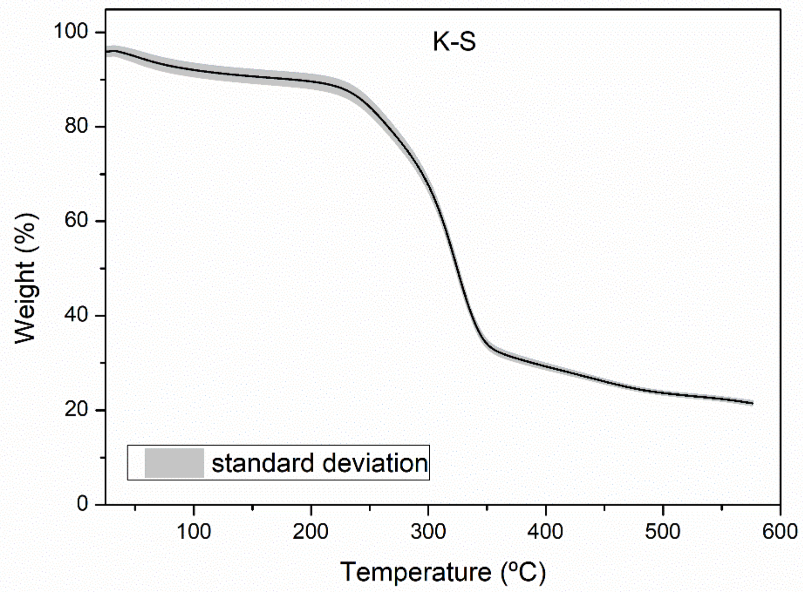

Figure S12. Average TGA curve with standard deviation for K-S (obtained with 3 specimens).

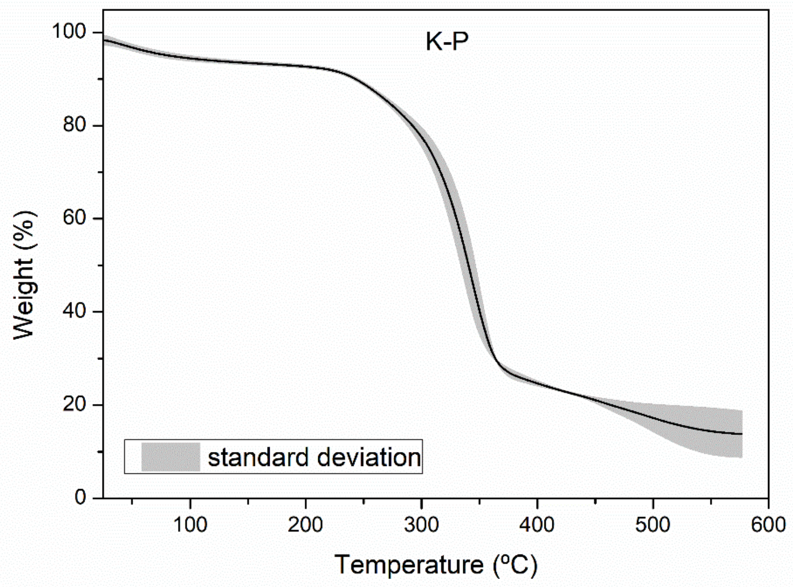

Figure S13. Average TGA curve with standard deviation for K-P (obtained with 3 specimens).

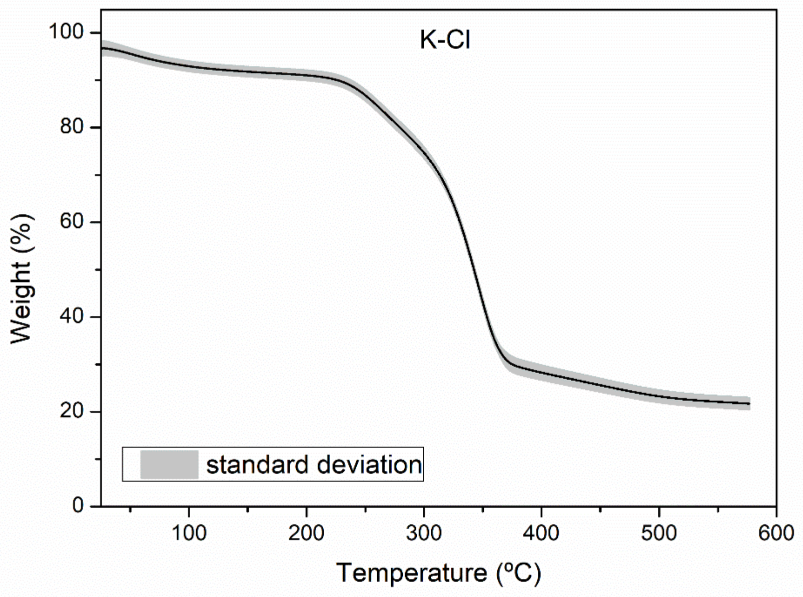

Figure S14. Average TGA curve with standard deviation for K-Cl (obtained with 3 specimens).

Table S1. Thermal properties obtained from average TGA curves for each sample (obtained with 3 freeze-dried specimens).

Sample	T_{max1} (°C)	Weight loss (%)	T_{max2} (°C)	Weight loss (%)	Char residue at 500 °C (%)
K-P	255.3 ± 2.0	11.9 ± 0.4	343.3 ± 8.0	53.3 ± 2.0	17.3 ± 2.9
K-S	261.8 ± 0.8	18.1 ± 1.2	329.3 ± 1.2	49.3 ± 0.6	24.1 ± 0.4
K-Cl	261.0 ± 2,3	15.5 ± 0.7	345.4 ± 0.2	52.7 ± 1.1	23.3 ± 1.3
